# Supplementary material for: Unveiling the Bioactive Potential of the Invasive Jellyfish Phyllorhiza punctata Through Integrative Transcriptomic and Proteomic Analyses
Source: Biomolecules. 2025 Aug 4;15(8):1121. doi: 10.3390/biom15081121 (PMC12383608; doi:10.3390/biom15081121)
Supplement: Supplementary file 1 [file biomolecules-15-01121-s001.zip › Suplementary Material/FileS3/PEPFOLD4/XP_065058963_11221/clusters.html]

 PEP-FOLD clusters


Download archive of all models  here   

|  |  |  |
| --- | --- | --- |
| # | file | sOPEP |
| # cluster 1 - Size: 2 |  |  |
|  | XP\_065058963\_11221-model1 | -47.8261 |
|  | XP\_065058963\_11221-model1.1 | -47.7966 |
| # cluster 2 - Size: 2 |  |  |
|  | XP\_065058963\_11221-model2 | -47.256 |
|  | XP\_065058963\_11221-model2.1 | -47.2134 |
| # cluster 3 - Size: 4 |  |  |
|  | XP\_065058963\_11221-model3 | -46.004 |
|  | XP\_065058963\_11221-model3.1 | -45.7093 |
|  | XP\_065058963\_11221-model3.2 | -44.1263 |
|  | XP\_065058963\_11221-model3.3 | -44.0112 |
| # cluster 4 - Size: 10 |  |  |
|  | XP\_065058963\_11221-model4 | -45.5012 |
|  | XP\_065058963\_11221-model4.1 | -44.4872 |
|  | XP\_065058963\_11221-model4.2 | -43.8055 |
|  | XP\_065058963\_11221-model4.3 | -43.7987 |
|  | XP\_065058963\_11221-model4.4 | -43.5417 |
|  | XP\_065058963\_11221-model4.5 | -43.1749 |
|  | XP\_065058963\_11221-model4.6 | -43.1475 |
|  | XP\_065058963\_11221-model4.7 | -43.0499 |
|  | XP\_065058963\_11221-model4.8 | -42.8499 |
|  | XP\_065058963\_11221-model4.9 | -42.8396 |
| # cluster 5 - Size: 10 |  |  |
|  | XP\_065058963\_11221-model5 | -45.375 |
|  | XP\_065058963\_11221-model5.1 | -45.312 |
|  | XP\_065058963\_11221-model5.2 | -45.2586 |
|  | XP\_065058963\_11221-model5.3 | -44.8757 |
|  | XP\_065058963\_11221-model5.4 | -44.7038 |
|  | XP\_065058963\_11221-model5.5 | -44.5818 |
|  | XP\_065058963\_11221-model5.6 | -44.5304 |
|  | XP\_065058963\_11221-model5.7 | -44.4405 |
|  | XP\_065058963\_11221-model5.8 | -43.8663 |
|  | XP\_065058963\_11221-model5.9 | -43.6674 |
| # cluster 6 - Size: 5 |  |  |
|  | XP\_065058963\_11221-model6 | -44.8716 |
|  | XP\_065058963\_11221-model6.1 | -44.3506 |
|  | XP\_065058963\_11221-model6.2 | -44.2504 |
|  | XP\_065058963\_11221-model6.3 | -43.4829 |
|  | XP\_065058963\_11221-model6.4 | -42.3942 |
| # cluster 7 - Size: 2 |  |  |
|  | XP\_065058963\_11221-model7 | -41.875 |
|  | XP\_065058963\_11221-model7.1 | -41.0931 |
| # cluster 8 - Size: 2 |  |  |
|  | XP\_065058963\_11221-model8 | -41.1835 |
|  | XP\_065058963\_11221-model8.1 | -41.0369 |
| # cluster 9 - Size: 3 |  |  |
|  | XP\_065058963\_11221-model9 | -41.1746 |
|  | XP\_065058963\_11221-model9.1 | -34.8554 |
|  | XP\_065058963\_11221-model9.2 | -34.6055 |
| # cluster 10 - Size: 2 |  |  |
|  | XP\_065058963\_11221-model10 | -40.4373 |
|  | XP\_065058963\_11221-model10.1 | -35.3452 |
| # cluster 11 - Size: 1 |  |  |
|  | XP\_065058963\_11221-model11 | -40.4153 |
| # cluster 12 - Size: 5 |  |  |
|  | XP\_065058963\_11221-model12 | -39.8604 |
|  | XP\_065058963\_11221-model12.1 | -38.8911 |
|  | XP\_065058963\_11221-model12.2 | -37.6241 |
|  | XP\_065058963\_11221-model12.3 | -37.1536 |
|  | XP\_065058963\_11221-model12.4 | -36.9048 |
| # cluster 13 - Size: 2 |  |  |
|  | XP\_065058963\_11221-model13 | -39.4963 |
|  | XP\_065058963\_11221-model13.1 | -35.8259 |
| # cluster 14 - Size: 2 |  |  |
|  | XP\_065058963\_11221-model14 | -39.0903 |
|  | XP\_065058963\_11221-model14.1 | -35.243 |
| # cluster 15 - Size: 2 |  |  |
|  | XP\_065058963\_11221-model15 | -38.8821 |
|  | XP\_065058963\_11221-model15.1 | -38.5786 |
| # cluster 16 - Size: 4 |  |  |
|  | XP\_065058963\_11221-model16 | -38.3727 |
|  | XP\_065058963\_11221-model16.1 | -37.4105 |
|  | XP\_065058963\_11221-model16.2 | -37.403 |
|  | XP\_065058963\_11221-model16.3 | -37.3283 |
| # cluster 17 - Size: 3 |  |  |
|  | XP\_065058963\_11221-model17 | -38.0454 |
|  | XP\_065058963\_11221-model17.1 | -36.9001 |
|  | XP\_065058963\_11221-model17.2 | -35.9964 |
| # cluster 18 - Size: 4 |  |  |
|  | XP\_065058963\_11221-model18 | -37.7822 |
|  | XP\_065058963\_11221-model18.1 | -35.8859 |
|  | XP\_065058963\_11221-model18.2 | -35.0745 |
|  | XP\_065058963\_11221-model18.3 | -34.9054 |
| # cluster 19 - Size: 2 |  |  |
|  | XP\_065058963\_11221-model19 | -37.0335 |
|  | XP\_065058963\_11221-model19.1 | -32.1388 |
| # cluster 20 - Size: 3 |  |  |
|  | XP\_065058963\_11221-model20 | -36.7459 |
|  | XP\_065058963\_11221-model20.1 | -35.7148 |
|  | XP\_065058963\_11221-model20.2 | -34.3775 |
| # cluster 21 - Size: 2 |  |  |
|  | XP\_065058963\_11221-model21 | -36.4444 |
|  | XP\_065058963\_11221-model21.1 | -34.9393 |
| # cluster 22 - Size: 1 |  |  |
|  | XP\_065058963\_11221-model22 | -36.4222 |
| # cluster 23 - Size: 2 |  |  |
|  | XP\_065058963\_11221-model23 | -36.4039 |
|  | XP\_065058963\_11221-model23.1 | -35.5639 |
| # cluster 24 - Size: 1 |  |  |
|  | XP\_065058963\_11221-model24 | -36.2314 |
| # cluster 25 - Size: 3 |  |  |
|  | XP\_065058963\_11221-model25 | -36.1336 |
|  | XP\_065058963\_11221-model25.1 | -35.3882 |
|  | XP\_065058963\_11221-model25.2 | -35.1182 |
| # cluster 26 - Size: 2 |  |  |
|  | XP\_065058963\_11221-model26 | -35.8298 |
|  | XP\_065058963\_11221-model26.1 | -35.6221 |
| # cluster 27 - Size: 1 |  |  |
|  | XP\_065058963\_11221-model27 | -35.7265 |
| # cluster 28 - Size: 2 |  |  |
|  | XP\_065058963\_11221-model28 | -35.4721 |
|  | XP\_065058963\_11221-model28.1 | -34.8285 |
| # cluster 29 - Size: 3 |  |  |
|  | XP\_065058963\_11221-model29 | -35.4556 |
|  | XP\_065058963\_11221-model29.1 | -35.1146 |
|  | XP\_065058963\_11221-model29.2 | -33.6213 |
| # cluster 30 - Size: 3 |  |  |
|  | XP\_065058963\_11221-model30 | -35.4119 |
|  | XP\_065058963\_11221-model30.1 | -35.3157 |
|  | XP\_065058963\_11221-model30.2 | -34.3937 |
| # cluster 31 - Size: 2 |  |  |
|  | XP\_065058963\_11221-model31 | -34.9295 |
|  | XP\_065058963\_11221-model31.1 | -33.7205 |
| # cluster 32 - Size: 1 |  |  |
|  | XP\_065058963\_11221-model32 | -34.7506 |
| # cluster 33 - Size: 1 |  |  |
|  | XP\_065058963\_11221-model33 | -34.6089 |
| # cluster 34 - Size: 1 |  |  |
|  | XP\_065058963\_11221-model34 | -34.4482 |
| # cluster 35 - Size: 1 |  |  |
|  | XP\_065058963\_11221-model35 | -34.2493 |
| # cluster 36 - Size: 2 |  |  |
|  | XP\_065058963\_11221-model36 | -34.0101 |
|  | XP\_065058963\_11221-model36.1 | -32.8398 |
| # cluster 37 - Size: 2 |  |  |
|  | XP\_065058963\_11221-model37 | -33.8082 |
|  | XP\_065058963\_11221-model37.1 | -33.1652 |
|  |  |  |
